# Supplementary figures and images for: Host Immunity Alters Community Ecology and Stability of the Microbiome in a Caenorhabditis elegans Model
Source: mSystems. 2021 Apr 20;6(2):e00608-20. doi: 10.1128/mSystems.00608-20 (PMC8561663; doi:10.1128/mSystems.00608-20)

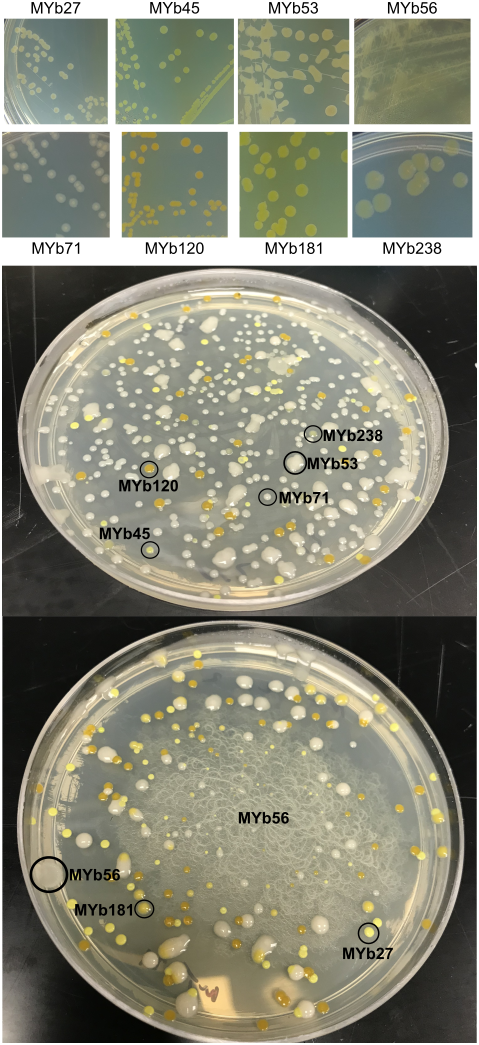

Supplement: FIG S1 [file msystems.00608-20-sf001.tif]

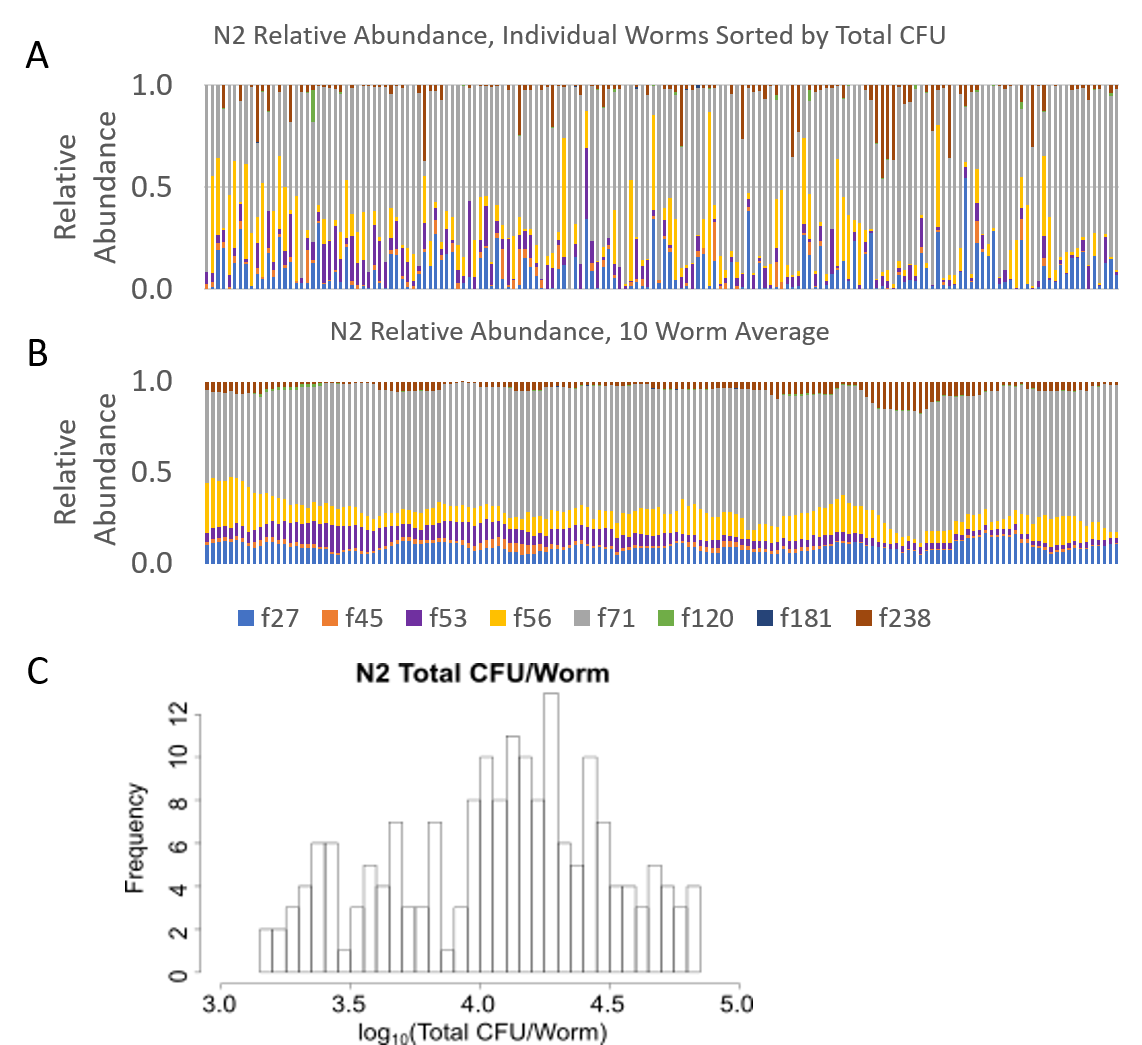

Supplement: FIG S2 [file msystems.00608-20-sf002.tif]

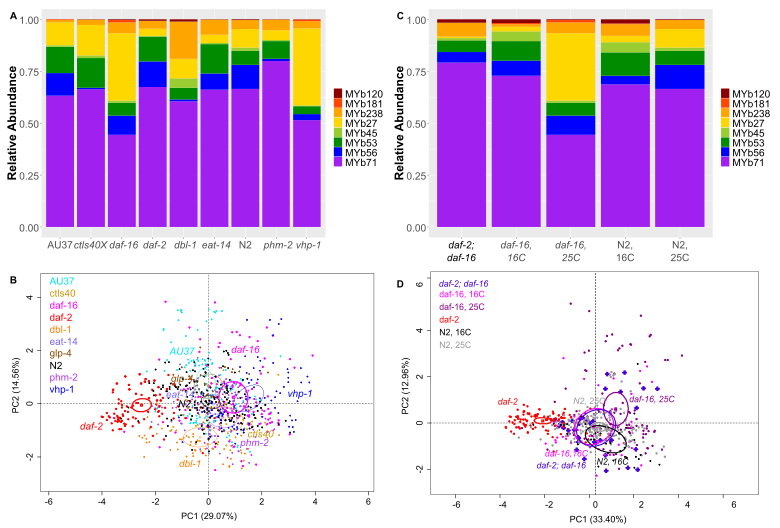

Supplement: FIG S3 [file msystems.00608-20-sf003.tif]

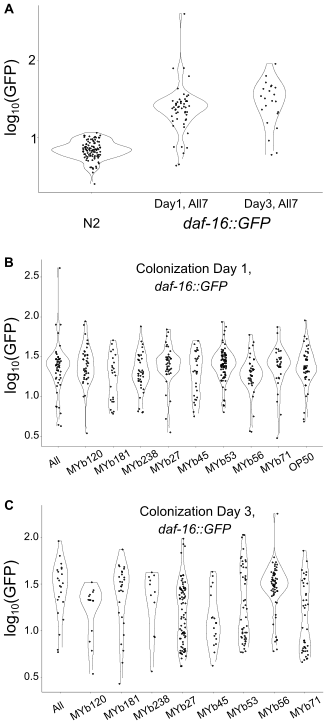

Supplement: FIG S4 [file msystems.00608-20-sf004.tif]

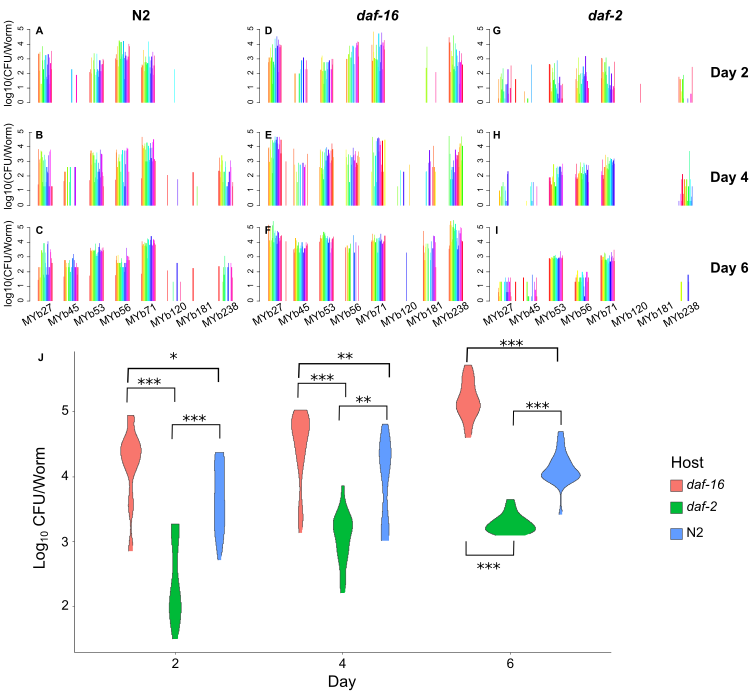

Supplement: FIG S5 [file msystems.00608-20-sf005.tif]

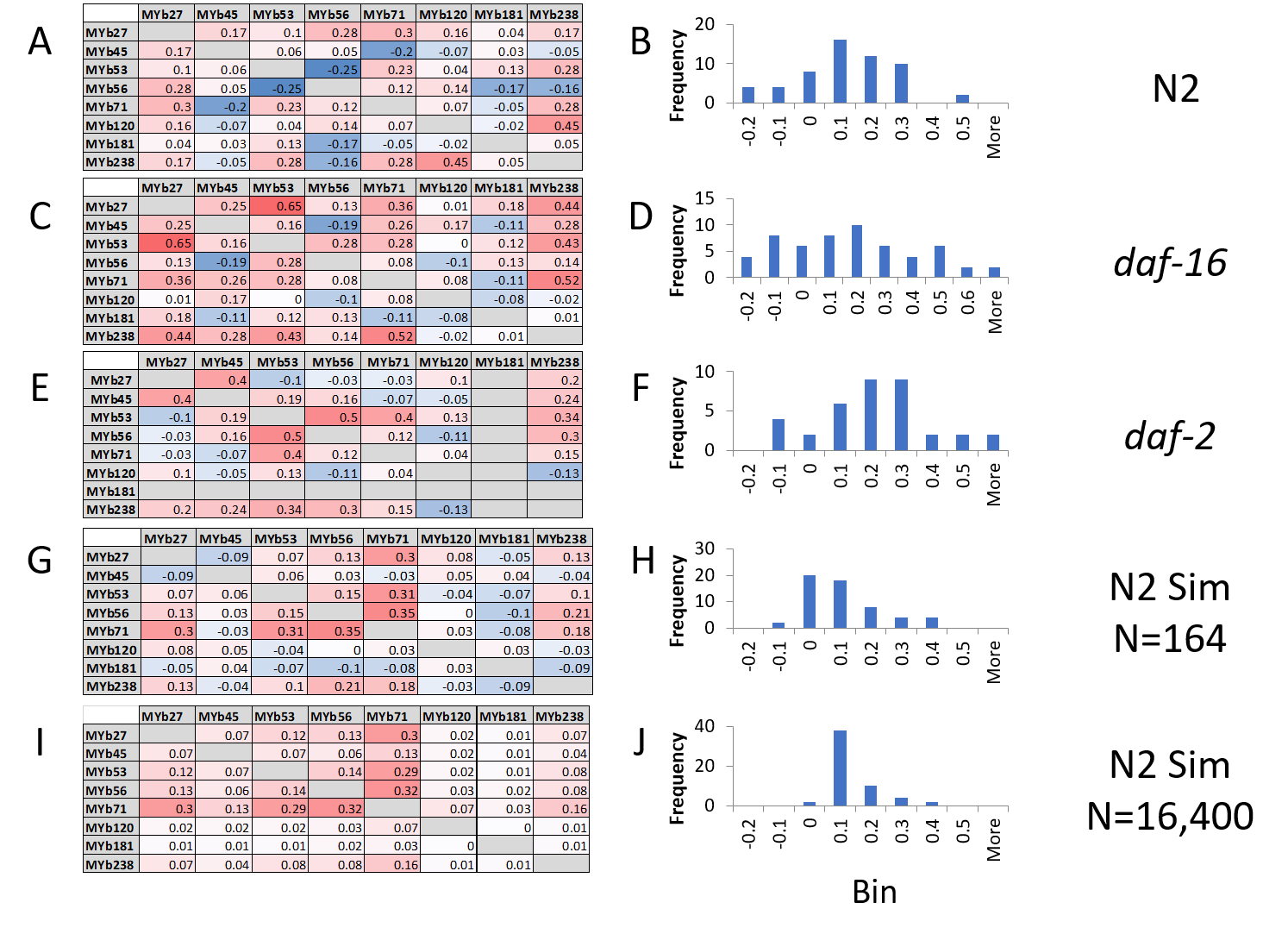

Supplement: FIG S6 [file msystems.00608-20-sf006.tif]

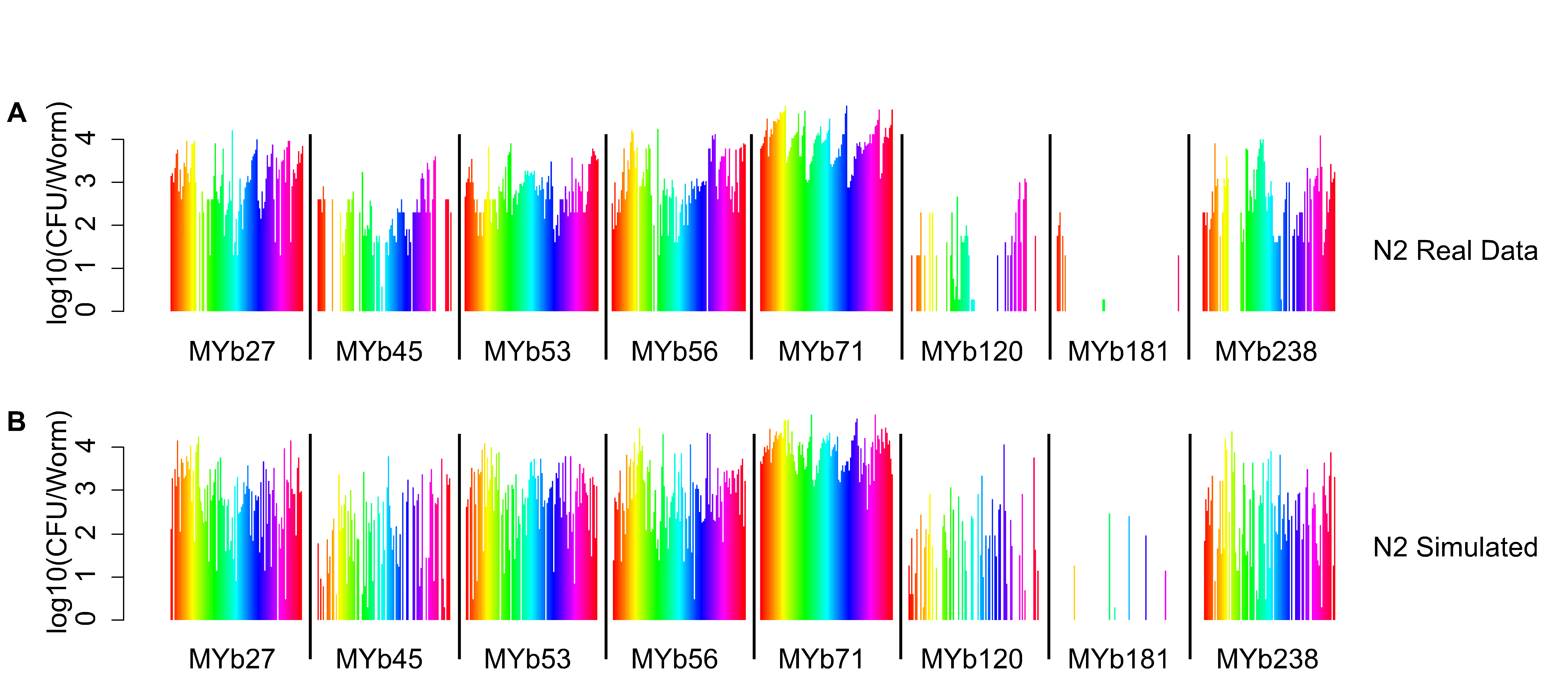

Supplement: FIG S7 [file msystems.00608-20-sf007.tif]

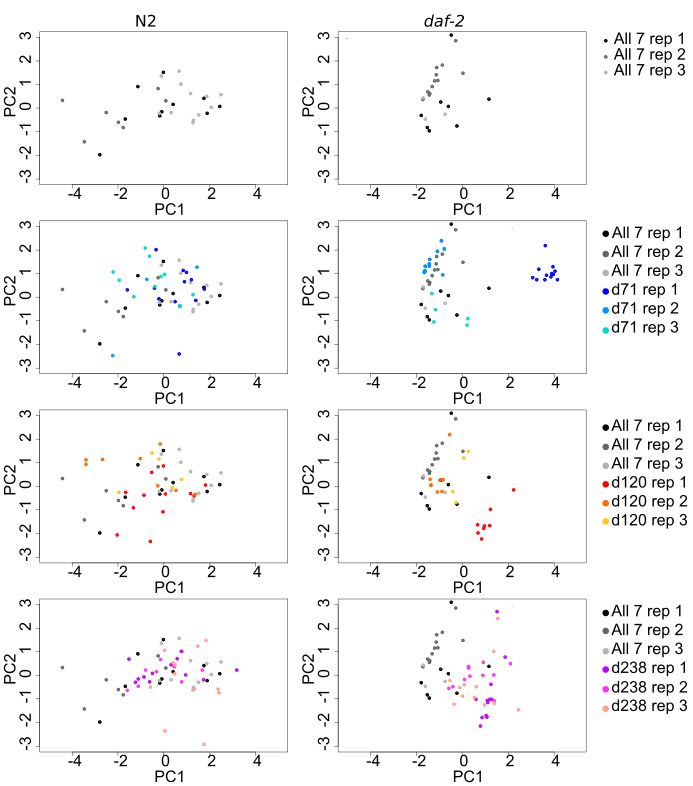

Supplement: FIG S8 [file msystems.00608-20-sf008.tif]
